# Mechanistic Potential and Therapeutic Implications of Cannabinoids in Nonalcoholic Fatty Liver Disease

**DOI:** 10.3390/medicines5020047

**Published:** 2018-05-28

**Authors:** Pratima Dibba, Andrew Li, George Cholankeril, Umair Iqbal, Chiranjeevi Gadiparthi, Muhammad Ali Khan, Donghee Kim, Aijaz Ahmed

**Affiliations:** 1 Division of Gastroenterology, Women & Infants Hospital/Warren Alpert School of Medicine, Brown University, Providence, RI 02905, USA; pratima_dibba@brown.edu; 2 Department of Medicine, Stanford University School of Medicine, Stanford, CA 94304, USA; andrewli@stanford.edu; 3 Division of Gastroenterology and Hepatology, Stanford University School of Medicine, Stanford, CA 94304, USA; georgetc@stanford.edu (G.C.); dhkimmd@stanford.edu (D.K.); 4 Department of Medicine, Mary Imogene Bassett Hospital, Cooperstown, NY 13326, USA; umairiqbal_dmc@hotmail.com; 5 Division of Gastroenterology and Hepatology, University of Tennessee Health Science Center, Memphis, TN 38163, USA; chirudoc@yahoo.com (C.G.); mkhan24@uthsc.edu (M.A.K.)

**Keywords:** nonalcoholic fatty liver disease, NAFLD, nonalcoholic steatohepatitis, NASH, cannabinoids, endocannabinoid system, endocannabinoid, exocannabinoid

## Abstract

Nonalcoholic fatty liver disease (NAFLD) is comprised of nonalcoholic fatty liver (NAFL) and nonalcoholic steatohepatitis (NASH). It is defined by histologic or radiographic evidence of steatosis in the absence of alternative etiologies, including significant alcohol consumption, steatogenic medication use, or hereditary disorders. NAFLD is now the most common liver disease, and when NASH is present it can progress to fibrosis and hepatocellular carcinoma. Different mechanisms have been identified as contributors to the physiology of NAFLD; insulin resistance and related metabolic derangements have been the hallmark of physiology associated with NAFLD. The mainstay of treatment has classically involved lifestyle modifications focused on the reduction of insulin resistance. However, emerging evidence suggests that the endocannabinoid system and its associated cannabinoid receptors and ligands have mechanistic and therapeutic implications in metabolic derangements and specifically in NAFLD. Cannabinoid receptor 1 antagonism has demonstrated promising effects with increased resistance to hepatic steatosis, reversal of hepatic steatosis, and improvements in glycemic control, insulin resistance, and dyslipidemia. Literature regarding the role of cannabinoid receptor 2 in NAFLD is controversial. Exocannabinoids and endocannabinoids have demonstrated some therapeutic impact on metabolic derangements associated with NAFLD, although literature regarding direct therapeutic use in NAFLD is limited. Nonetheless, the properties of the endocannabinoid system, its receptors, substrates, and ligands remain a significant arena warranting further research, with potential for a pharmacologic intervention for a disease with an anticipated increase in economic and clinical burden.

## 1. Introduction

The predicted clinical burden of nonalcoholic fatty liver disease (NAFLD) is substantial, with over 12 million incident cases expected in the United States and 600,000 in Europe [1]. The economic burden is predicted to be $103 billion and €35 billion in the United States and Europe, respectively [1]. These burdens can largely be attributed to a lack of availability of treatment and chemopreventive agents for NAFLD or NAFLD-associated hepatocellular carcinoma (HCC) [2]. Lifestyle modifications (including weight loss, dietary changes, and exercise) have been the mainstay of therapy, with metformin, statins, S-adenosylmethonine, vitamin replacements, and surgical resection of early stage nonalcoholic steatohepatitis-related hepatocellular carcinoma (NASH-HCC) having potential therapeutic implications. However, other therapeutic modalities are being studied. Of note, the endocannabinoid system, inclusive of its receptors, substrates, and ligands, has been studied for the past decade as it has mechanistic and therapeutic implications in NAFLD [3].

## 2. Definition of Nonalcoholic Fatty Liver Disease

NAFLD is defined as hepatic steatosis, diagnosed by histologic or radiographic evidence, for which no other etiologies (i.e., significant alcohol consumption, steatogenic medication use, hereditary disorders) are responsible [4]. It is characterized by the presence of fat accumulation (i.e., steatosis) in more than 5% of hepatocytes as identified by histology, proton density fat fraction, or magnetic resonance imaging [5]. Nonalcoholic fatty liver disease can be further categorized into nonalcoholic fatty liver (NAFL) and nonalcoholic steatohepatitis (NASH) based on histologic findings [4]. NAFL and NASH both histologically demonstrate the presence of hepatic steatosis, whereas NASH is more specifically distinguished by inflammation with hepatocyte injury (ballooning) with or without fibrosis [4].

## 3. Epidemiology of NAFLD

The prevalence of NAFLD varies based on the population studied and definition used [4]. Two studies utilizing liver donors estimated the global prevalence to be between 20% and 51% [4]. Studies utilizing evidence of disease diagnosed by ultrasound estimated the prevalence to be between 17% and 46% [4]. The prevalence of NAFLD in patients without metabolic risk factors remains as high as 16% [6]. The prevalence of NAFLD ranges between 6% and 35% worldwide, with wide variations inclusive of high prevalence in Europe [4,7]. The European Association for the Study of Liver (EASL) reports prevalence as high as 46% worldwide [6]. Nonetheless, NAFLD is the most common liver disease globally [4]. The incidence of NASH-related HCC ranges between 2.4% and 12.8%, primarily in those with advanced fibrosis or cirrhosis [8]. A study by Wong et al. analyzing liver transplant recipients between 2002 and 2012 identified NASH as the second leading cause of HCC-related liver transplantation [9].

## 4. Demographics and Risk Factors Associated with NAFLD

Mexican American ethnicity is associated with a higher risk of NAFLD, although this risk factor, along with male gender, demonstrated protection against advanced fibrosis within a cohort of NAFLD patients [10]. Lower education was identified as a risk factor for advanced fibrosis in the same cohort [10]. Prevalence is also noted to be higher in Americans of European descent and African Americans [11]. Risk factors associated with NAFLD can be found in Table 1. Risk factors agreed upon by EASL, the Asian-Pacific Working Party for NAFLD (APWP-NAFLD), the Chinese Liver Disease Association (CLDA), the Italian Association for the Study of Liver (IASL), and the American Gastroenterological Association (AGA)–American Association of the Study of Liver Disease (AASLD)–American College of Gastroenterology (ACG) include manifestations of insulin resistance, such as metabolic syndrome, type 2 diabetes mellitus, obesity, and dyslipidemia [12]. Emerging risk factors associated with NAFLD include polycystic ovary syndrome, hypothyroidism, obstructive sleep apnea, hypopituitarism, hypogonadism, and pancreatico-duodenal resection [4]. There are at least two genes and several variants that have been implicated as risk factors for susceptibility to NAFLD, whose presence varies across ethnic groups [11]. The literature supports the association of dietary composition and physical activity with NAFLD, with evidence of increased consumption of processed foods, foods with high salt/fat content or corn syrup, low nutrient/high sodium/high fat foods, low physical activity, and prolonged sitting times demonstrated in patients with NAFLD [11].

Risk factors identified in the progression of NASH to HCC include chronic systemic inflammation, increased lipid storage, lipid toxicity, genetic polymorphism, increased iron absorption, intestinal flora dysregulation, elevated lipopolysaccharide levels, insulin resistance, advanced age, concomitant alcohol use, and increased insulin growth factor levels [2].

## 5. Natural History of NAFLD

Patients with NAFL follow an insidious course with a lower likelihood of progression to cirrhosis, whereas patients with NASH can more commonly and rapidly exhibit progression to cirrhosis [4,13]. Patients with NASH can progress from fibrosis to cirrhosis with risk of development of HCC [2]. However, recent literature suggests that NAFLD and NASH can present with HCC without histologically progressing to cirrhosis [2]. Although the most common cause of death among all patients with NAFLD, including those with NAFL and NASH, is cardiovascular disease, liver disease and cancer are among the leading causes of death [5,14]. NAFLD-HCC has been demonstrated to have a higher tumor burden and greater aggressiveness versus HCC related to hepatitis C virus (HCV), resulting in decreased overall survival in the former group [15]. Those with NASH are at increased risk of cirrhosis and liver-disease related death [16,17].

## 6. Pathophysiology of NAFLD

Insulin resistance, a hallmark of physiology underlying NAFLD, is hypothesized as the cause of fat accumulation within the liver along with other etiologies of hepatic steatosis, including excess intracellular fatty acids, oxidant stress, adenosine triphosphate depletion, and mitochondrial dysfunction [18]. Hepatic steatosis is a result of the inability of the liver to oxidize or export free fatty acids that have been delivered to or synthesized by the liver via adipose tissue lipolysis, de novo lipogenesis, or dietary intake [6,19]. Additional lipid metabolites may also directly cause cell injury and interfere with the ability of insulin to phosphorylate insulin receptor substrate-2, resulting in insulin resistance [6]. More recent studies have correlated adipose tissue dysfunction with NAFLD as a pathogenic mechanism in the disease. While several risk factors for NAFLD have been proposed (Table 1), including obesity, adipose tissue itself has been shown to function in NAFLD by multiple mechanisms as an independent organ [6]. Visceral adipose tissue, in particular, expands in response to excess nutrient intake and accumulation of triglycerides, which, in turn, results in adipocyte hypertrophy associated with increased pro-inflammatory markers and adipokines (i.e., adinopectin, leptin, TNFα, and IL-6) [6]. Visceral adipose tissue can also progressively fibrose, limiting the amount of stored fat and causing ectopic deposition of fat in the liver [6]. Cimini et al. propose that vitamin D may play a role in the pathogenesis of NAFLD based on its association with obesity and an unfavorable metabolic profile as suggested in the existing literature [6]. Genetic modifiers have been associated with susceptibility to NAFLD via the presence of higher liver fat content, namely PNPLA3 (encoding palatin-like phospholipase domain-containing protein 3) and TM6SF2 (encoding transmembrane 6 superfamily member 2) [5,11].

## 7. The Mechanistic Role of the Endocannabinoid System in NAFLD

The role of cannabinoids has yet to reveal definite therapeutic properties in the treatment of NAFLD. Cannabinoids (particularly, daily cannabis use) have been previously been identified as an independent predictor of fibrosis and steatosis severity, although in the context of chronic HCV [20]. Additionally, active marijuana use in a nationally representative cohort was associated with a protective effect against NAFLD independent of metabolic risk factors [21].

The endocannabinoid system, within which cannabinoids in cannabis function, has potential mechanistic and therapeutic roles in NAFLD. Within the endocannabinoid system, two specific G-protein receptors have been identified that may play a role in liver disease [19]. Cannabinoid receptor 1 (CB1) and cannabinoid receptor 2 (CB2) are bound by ligands called endocannabinoids which are highly lipophilic [19]. CB1 is predominant in the brain and expressed in peripheral tissues at lower levels, functioning primarily in the psychotropic and behavioral effects elicited by cannabinoids [19]. CB2 is expressed primarily in the peripheral tissue with low-level activity detected in the central nervous system, functioning rather in the modulation of innate immunity and bone mass with some antitumor properties [19]. In particular, CB1 has been detected in endothelial cells and hepatocytes and CB2 has been identified in Kupffer cells [19]. Because of the metabolic effects of endocannabinoids, studies have investigated their possible utility in liver disease [21]. Endocannabinoids that have been extensively studied include anandamine (arachidonoyl ethanolamide, AEA) and 2-arachidonoyl glycerol (2-AG), which are synthesized on demand and activate CB1 and CB2 [19]. 2-AG was found to have a positive correlation with liver fat content in overweight women with NAFLD, suggesting that 2-AG may increase de novo lipogenesis [22]. The synthesis of 2-AG, AEA, and other endocannabinoids is increased by steatogenic agents, such as a high-fat diet, ethanol, and possibly obesity, potentiating the activity of CB1 receptors [23]. CB1 receptor activity upregulation has been hypothesized to lead to increased de novo fatty acid synthesis and decreased fatty acid oxidation [23]. It has also been hypothesized that CB1 upregulation increases lipogenic gene expression, activates lipoprotein lipase in adipose tissue, and decreases secretion of triglyceride-rich very-low-density lipoprotein [21]. The exocannabinoids, which are major components of cannabis, that have been studied include tetrahydrocannabinol (THC), tetrahydrocannabivarin (THCV), and cannabidiol (CBD) [24,25]. THC particularly acts on CB1, CB2, transient receptor potential cation channel subfamily C member 1, and the G-protein-coupled receptors GPR56 and GPR11 [24]. Recent literature suggests that THC increases AEA and 2-AG in hepatocytes by competing with them in binding to fatty acid binding protein-1, making them less available for degradation and hydrolysis [26]. THCV, an analog of THC, can act as a CB1/CB2 agonist in low doses and/or a CB1/CB2 neutral antagonist in high doses; however, further investigation is warranted as alternate studies show that isomers of THCV can induce agonist effects independently [27,28].

## 8. Therapeutic Potential for the Endocannabinoid System in NAFLD

The pharmacologic modulation of the endocannabinoid receptors has been studied in clinical settings concerning obesity, and secondary to their presence within the hepatic system, they have been additionally studied in NAFLD [19]. Increased endocannabinoid tone has been demonstrated in the setting of liver injury, with increases in endocannabinoids correlating to the nature of the injury [3]. Antagonism of CB1 receptors has been demonstrated to reduce food intake and increase energy expenditure in animal studies, resulting in an improvement in the metabolic syndrome [19]. In fact, prior studies showed that mice deficient in CB1 were resistant to diet-induced obesity, and increases in de novo lipogenesis in mice consuming a high-fat diet were inhibited by CB1 antagonism [29]. Jourdan et al. demonstrated that reduction in CB1 activity in visceral fat was associated with normalization of adipocyte metabolism in animal models as well [30]. Similar findings in 2008 by Osei-Hyiaman et al. were coupled with findings of resistance within obese mice to steatosis, dyslipidemia, and insulin resistance [31]. The results of this study showed that AEA activates a pathway within the liver that participates in the development of diet-induced obesity and hepatic steatosis [26]. Due to its association with a reduction of body weight, waist circumference, triglyceride levels, and hyperinsulinemia, the CB1 receptor antagonist Rimonabant was studied for its effects on hepatic steatosis [32,33,34,35]. Rimonabant reduced hepatomegaly and hepatic steatosis, reduced markers of liver damage, and reduced levels of hepatic TNFα, suggesting a reduction in hepatic inflammation [35]. Such findings implied a reduction in insulin resistance within the liver with slowed progression of hepatic steatosis to fibrosis and cirrhosis [35]. Although this drug was initially approved for use within the United States, it was withdrawn after being found to increase the risk of central nervous system toxicity, particularly anxiety and depression [36,37]. An alternative non-brain-penetrant CB1 antagonist, AM6545, was studied thereafter, demonstrating reduced food intake without malaise, improved glycemic control, improved dyslipidemia, and reversal of hepatic steatosis [38,39]. Proposed mechanisms in the improvement of metabolic factors by AM6545 include an increase in adiponectin and a reduction of TNFα and leptin levels [40]. JD5037, a peripherally-restricted CB1 inverse agonist, has also been demonstrated to reduce food intake, body weight, and adiposity in animal studies without established psychiatric side effects, showing improved potential for a therapeutic role in hepatic steatosis [41]. The role of CB2 agonism versus antagonism is conflicting in the current literature. Although CB2 has been established as antisteatogenic in alcohol-mediated liver disease by reducing steatogenic cytokines in CB2-stimulated Kuppfer cells, its role in NAFLD is controversial [42]. In previous animal studies examining the role of CB2 receptors in adipose tissue inflammation, CB2 agonists, such as JWH-133, increased obesity-associated inflammation, insulin resistance, and hepatic steatosis [43]. Conversely, CB2-deficient mice demonstrated improvement of steatosis and insulin resistance despite increases in food intake and body weight with age [44]. A study by De Godartti et al. supports these findings with the demonstration of an increase in the degree of steatosis by CB2 agonists [45]. Other studies suggest that CB2 agonism in rats improved glucose tolerance, while CB2 antagonists, such as AM630, worsened glucose tolerance [46]. Recent studies suggest that CB2 may play a role in reducing acute liver injury [47,48]. CB2 receptors activated by 2-AG have also been postulated to allow for hepatocyte survival and regeneration following acute insult, suggesting that they may be antifibrinogenic [19]. However, this hypothesis has been questioned in a study conducted by Louvet et al. [49]. Studies additionally suggest that CB1 receptors, whose antagonism may be antifibrinogenic, may also promote liver regeneration via activation by AEA [42,50].

A summary of agonism and antagonism/deficiency of CB1 can be found in Table 2. The agonism and antagonism/deficiency of CB2 has been excluded due to conflicting findings regarding its role in NAFLD. Exocannabinoids (i.e., THC, THCV, CBD), in addition to endocannabinoids, can mediate effects via cannabinoid receptors as aforementioned [24,25]. Some studies suggest that CBD and THCV improve insulin resistance and reduce accumulated lipid levels in a model of hepatosteatosis, suggesting potential therapeutic effects in NAFLD [25]. CBD has demonstrated a reduction in total cholesterol with increases in high-density lipoprotein cholesterol as well as a reduced liver triglyceride concentration [27]. THCV causes hypophagia and weight loss [28]. One study showed that it additionally demonstrated reduced body fat content, increased energy expenditure, reduced fasting insulin and a 30-min insulin response to oral glucose tolerance testing, and reduced liver triglycerides similar to the CB1 antagonist AM251 [51]. Its positive effects on glycemic control were reiterated by a study done by Jadoon et al. [27]. Studies also demonstrate that THC provides protection against ischemic-reperfusion injury within the liver at low doses [52]. With such therapeutic effects on metabolic risk factors associated with NAFLD, the therapeutic role of cannabis use is being investigated in the setting of NAFLD. A study by Adejumo et al. demonstrated that cannabis use was associated with a lower prevalence of NAFLD [53]. There remains a paucity of literature regarding the direct therapeutic benefit of exocannabinoids in NAFLD.

## 9. Conclusions

Due to the anticipated rise of the economic and clinical burden of NAFLD, which is now the most common liver disease globally, and the potential for progression or occurrence of hepatocellular carcinoma, newer modalities of therapy are being investigated [1,4]. While lifestyle modifications have been the mainstay of therapy, recent investigation of the role of the endocannabinoid system has revealed mechanistic properties specific to hepatic steatosis. Due to the presence of such mechanistic properties, therapies based on the pharmacomodulation of cannabinoid receptors have shown promising and informative results. Although CB2 agonism and antagonism have shown conflicting results regarding their role in NAFLD, CB1 antagonism has demonstrated therapeutic benefits especially when central nervous system penetration has been minimized or eliminated [35,37,54]. Literature regarding the therapeutic benefits of cannabinoids, although promising, is limited and warrants further investigation.

## Figures and Tables

**Table 1:** Risk Factors Associated with nonalcoholic fatty liver disease (NAFLD) [4].

Conditions with Established Association	Conditions with Emerging Association *
**Obesity**	Polycystic ovary syndrome
**Type 2 diabetes mellitus**	Hypothyroidism
**Dyslipidemia**	Obstructive Sleep apnea
**Metabolic syndrome ** **	Hypopituitarism
	Hypogonadism
	Pancreato-duodenal resection

* A few studies have suggested that individuals with type1 diabetes have increased prevalence of hepatic steatosis based on liver imaging, but there is limited histological evidence. ** The Adult Treatment Panel III (the National Cholesterol Education Program Expert Panel on the detection, evaluation, and treatment of High Blood Cholesterol in Adults) clinical definition of the metabolic syndrome requires the presence of three or more of the following features: (1) a waist circumference greater than 102 cm in men or greater than 88 cm in women; (2) a triglyceride level of 150 mg/dL or greater; (3) a high-density lipoprotein (HDL) cholesterol level of less than 40 mg/dL in men and less than 50 mg/dL in women; (4) a systolic blood pressure of 130 mm Hg or greater or a diastolic pressure of 85 mm Hg or greater; and (5) a fasting plasma glucose level of 110 mg/dL or greater.

**Table 2:** Agonism and Antagonism and/or Deficiency of CB1.

Cannabinoid Receptor 1 (CB1) Agonism	CB1 Antagonism and/or Deficiency
Increased rate of de novo fatty acid synthesis (via upregulation) [21,23]	Reduce food intake (also via antagonism by AM6545 and inverse agonism by JD5037) [19,38,39,41]
Decreased fatty acid oxidation (via upregulation) [21,23]	Increased energy expenditure (via antagonism) [19]
Increased lipogenic gene expression (via upregulation) [21]	Improved metabolic syndrome [19]
Activation of lipoprotein lipase in adipose tissue (via upregulation) [21]	Resistance to diet-induced obesity (via deficiency) [29]
Decreased secretion of triglyceride-rich very low density lipoprotein (via upregulation) [21]	Inhibition of increased de novo lipogenesis (via antagonism) [24]
Liver regeneration [48]	Normalization of adipocyte metabolism (via reduction in activity) [30]
	Resistance to steatosis, dyslipidemia and insulin resistance [31]
	Reduction in markers of liver damage (via antagonism by Rimonabant) [35]
	Reduction of hepatic TNFα (via antagonism by Rimonabant) [35]
	Reduction in insulin resistance (via antagonism by Rimonabant) [35]
	Slowed progression of hepatic steatosis to fibrosis and cirrhosis (via antagonism by Rimonabant) [35]
	Improved glycemic control (via antagonism by AM6545) [38,39]
	Improved dyslipidemia (via antagonism by AM6545) [38,39]
	Reversal of hepatic steatosis (via antagonism by AM6545) [38,39]
	Reduction of body weight and adiposity (via inverse agonism by JD5037) [41]
	Antifibrinogenic [48]
